# Behavior of Silver Species in Soil: Ag Nanoparticles vs. Ionic Ag

**DOI:** 10.3390/molecules29235531

**Published:** 2024-11-22

**Authors:** Joanna Kyziol-Komosinska, Agnieszka Dzieniszewska, Justyna Czupioł

**Affiliations:** Institute of Environmental Engineering, Polish Academy of Sciences, 34 M Skłodowska-Curie St., 41-819 Zabrze, Poland; joanna.komosinska@ipispan.edu.pl (J.K.-K.); justyna.czupiol@ipispan.edu.pl (J.C.)

**Keywords:** adsorption, clay minerals, Fe (oxyhydr)oxides, oxidative dissolution, sequential chemical extraction, silver forms, soil organic matter

## Abstract

Silver nanoparticles are one of the most commonly used forms of silver (Ag) in nanotechnology applications due to their antibacterial properties and electrical and thermal resistance. The increasing production and use of products containing nanoparticles has led to their release into and contamination of soil and water. This review summarizes the literature on the fate, behavior (adsorption/desorption, precipitation/oxidative dissolution, transformation), and transport/mobility of Ag forms in soils (Ag^+^ ions and Ag nanoparticles—AgNPs). The behavior of Ag^+^/AgNPs in soil is a complex process. It depends on many factors, including the characteristics of the Ag forms (ions, nanoparticle size, ligand type used for coating, surface charge, initial Ag concentration), the soil properties (organic matter and clay mineral content, textural properties, point of zero charge, cation exchange capacity, surface functional groups), and the solute properties (pH–Eh, ionic strength, cation type, oxygen content). The binding of Ag^+^ and AgNPs is significantly positively correlated with Al/Fe/Mn oxide and SOM content and depends on the surface charge of the minerals and CEC, which controls adsorption processes. Very important parameters to consider are the pH and Eh of the solution, which determine the durability of the ligands, the aggregation rate and the oxidation process of AgNPs, as well as the presence of sulfide and chloride and the Cl/Ag ratio, which determine the stability/mobility of Ag. Since AgNPs can be oxidized to Ag^+^ ions during their life cycle, it is necessary to consider the behavior of both forms of Ag in soils. Understanding the transport and behavior of Ag in soil is essential for the environmental risk assessment and management of wastes containing Ag.

## 1. Introduction

**Figure 1:**
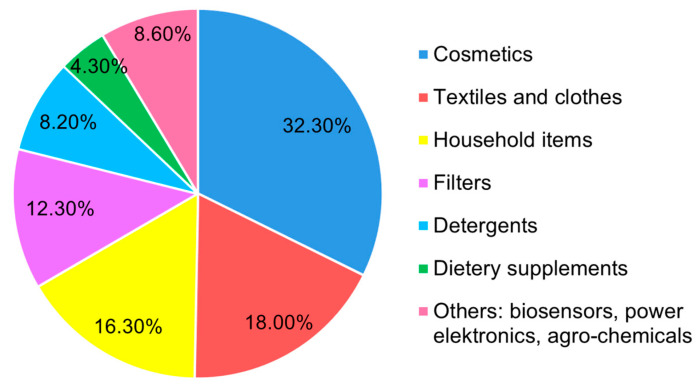
Main sources of AgNPs released into the environment [14].

Some metals and their oxides are currently used in functionalized products in nanoparticle (NP) form. The use of nanoparticles and nanomaterials in new products is growing rapidly on the market. Among the most popular and most widely produced are silver nanoparticles (AgNPs) [1,2,3]. At the moment, the list of 5367 known nanoproducts includes 1093 products that contain Ag [4,5] and is increasing. AgNPs, in addition to Ag^+^ ions, have been incorporated into numerous consumer products (Figure 1), such as textiles, household appliances, food containers, nanofunctionalized plastics, paints, cosmetics, personal care products, and nursery and medical products, and are used for disinfection of swimming pool water due to their antimicrobial and disinfecting properties, such as against Gram-positive and Gram-negative bacteria, viruses, fungi, and various drug-resistant strains [6,7,8,9,10,11]. AgNPs are used also in photonics, medicine, biotechnology, and bioengineering [12] due to unique optical properties, mechanical strength, and high electrical and thermal conductivity [13].

Increased production, distribution, use, and end-of-life products result in increasing amounts of AgNPs in both domestic and industrial wastewater and in landfill waste. The treatment of wastewater at wastewater treatment plants (WWTPs) produces sludge that is enriched in Ag, as indicated by Ag concentrations at the plant inlet and in the treated effluent ranging from 1.8 to >100 µg/L and <1 µg/L, respectively [15,16]. Although the majority (>98%) of AgNPs are retained in the sediment in the insoluble form of Ag_2_S or AgCl, the remainder (<2%) containing smaller AgNPs (20 nm) can be transported to the soil and aquatic environment [15,17]. Assuming that about 18% of sewage sludge is used as fertilizer in agriculture in the EU [18], the content of AgNPs in soils and groundwater is a real problem. AgNPs enter the water and soil via waste leachate from landfills, irrigation with wastewater, fertilization with sewage sludge from WWTPs, dry and wet atmospheric deposition, rainwater runoff, and erosion [19,20,21,22,23,24,25]. Other sources of elevated Ag content in soil and water are natural leaching from bedrock and mining activities [26]. Therefore, the behavior of AgNPs, including retention, transformation, mobility, and release into ecosystems, should be well understood for environmental risk assessment and management of wastes containing AgNPs [22,27,28,29,30], as well as for safety and assessment of environmental toxicity.

In soils, AgNPs can undergo transformations such as oxidative dissolution, and therefore the behavior of AgNPs and ionic Ag^+^ should be considered in parallel. The behavior of these species is different due to the charge and size of the nanoparticle/ion.

The aim of this review is to present the behavior of AgNPs as well as that of Ag^+^, which is formed by oxidation of nanoparticles and characterized by different properties from AgNPs. Nanoparticle size, structure, and surface charge, soil properties (organic matter and clay mineral content, textural properties, zero-charge point, pH, cation exchange capacity, surface functional groups), and solution properties (pH–Eh, ionic strength, cation type, oxygen content) are considered. The results of studies carried out under both static and dynamic conditions and the susceptibility of Ag retained by the minerals to release are presented and compared. Information on the adsorption and transport of AgNPs studied under dynamic conditions is still scarce, as many studies have been carried out on pure quartz [31,32], which is not representative of nanoparticle transport in natural soils [33]. We have pointed to the lack of standardized methods for studying the interaction of AgNPs with soils, as well as the different adsorption parameters used in different studies on Ag uptake by soils, making direct comparisons between different studies difficult [34]. To the best of our knowledge, this is the most comprehensive description of the behavior of Ag forms in soils.

## 2. Properties of Silver and Its Chemical Forms

Silver (Ag) is a rare and non-essential transition metal. Ag occurs in several forms: metallic (Ag^0^), ionic (Ag^+^, Ag^2+^, Ag^3+^), and as nanoparticles of 1 and 100 nm in diameter (AgNPs), and can be highly toxic to a wide range of biota, even at trace levels [35,36].

According to the U.S. Environmental Protection Agency (US EPA), Ag is on the list of priority toxic pollutants and Ag in drinking water is classified as a secondary contaminant at a concentration of 0.1 mg/L, with no distinction between AgNPs and Ag(aq) [37]. The measured concentrations of Ag in natural and contaminated water are in the range of nanograms per liter [38].

Its geochemical background is 0.038 mg/kg [39] and Ag content in surface soils ranges from <0.01 to 5 mg/kg in organic soils, with a mean of <1 mg/kg [40,41].

### 2.1. Ionic Form of Silver

Ag^+^ is the most common oxidation states in environment and stable in the water [42,43]. The Ag^+^ with ionic radius of 1.26 Å, can be incorporated into the interlayers of illitic or vermiculitic clays like lithophilic K^+^ [44]. Ag^+^ ions are mild oxidants and are weakly hydrolyzed in solution. They react with negatively charged ions or ligands, such as halides (Cl^−^, Br^−^ and I^−^) and sulfates (SO_4_^2−^), to form poorly soluble compounds or soluble multi-halide complexes such as AgCl_4_^3−^, AgCl_2_^−^, and AgCl_3_^2−^ [45,46]. Typical concentrations of halides or sulfates in soil solution may be too low for Ag precipitation. Ag^+^ ions have a strong affinity for sulfide ligands [47] and form highly insoluble Ag_2_S, with K_sp_ = 6 × 10^−51^ making them less toxic than Ag^+^ and AgNPs. Ag^+^ ions also form strong complexes with thiol (–SH) ligands [35].

### 2.2. Silver Nanoparticles (AgNPs)

The properties and behavior of AgNPs in water/soil depend on the following [48,49,50,51,52]:

- Nanoparticle size [53] and shape, e.g., spheres, rods, cubes, wires, and triangles [13,43];
- Coating types and kind of surface charge: (i) uncoated AgNPs (bare), (ii) modified with capping agents, such as ligands (citrate (CIT), tannic acid, sodium borohydride (NaBH_4_), methionine, thiosulfate), and (iii) modified with polymers (sodium polyacrylate, polyvinylpyrrolidone (PVP), polyethylene glycol, polyoxyethylene glycerol trioleate, branched polyethyleneimine (BEPI), polyoxyethylene (20) sorbitan mono-laurate (Tween 20), bovine serum albumin (BSA)) [54];
- Methodology used for the synthesis of AgNPs and their incorporation in products, such as pad–dry–cure, dip-coating, spin-coating, electroless deposition, thermo-synthesizing, spraying, sol–gel, microwave-assisted deposition, and ultrasound-assisted deposition [55,56];
- Initial Ag amount and fabric quality [57].

#### 2.2.1. Effect of Capping Agent on Behavior of AgNPs

In the soil–water environment, AgNPs may: (i) remain in soil solution, (ii) aggregate or agglomerate, (iii) dissolve, or (iv) react with various soil components [42,45].

The ability to aggregate (homoaggregate (between AgNPs) or heteroaggregate (between AgNPs and soil surface)) is based on colloidal interactions and van der Waals attraction and/or electrostatic repulsion, is described by the Derjaguin–Landau–Verwey–Overbeek (DLVO) theory, and depends on the coating type, charge at the nanoparticle surface, and properties of the solution [58]. Aggregation reduces the potential transport of AgNPs in soil/water because smaller particles move faster [59]. The aggregation rate increases with increasing the point of zero charge (pH_PZC_) [60,61] due to a decrease in the electrostatic repulsive forces between the nanoparticles.

Capping agents prevent aggregation and stabilize the particles [34,62,63], and can help control particle shape, reduce particle growth [42,43,64], and prevent oxidative dissolution.

The most commonly used capping agents for AgNPs, such as CIT, NaBH_4_, BPEI, and PVP [58], can significantly modify their surface chemistry (charge and magnitude) by generating positive or negative charges on the surface of AgNPs and changing the zeta potential [46,65]. For example, the zeta potential of bare AgNPs is −36 mV. CIT coating reduces particle zeta potential to −59 mV, while BPEI results in positively charged particles [1]. On the other hand, information on the charge of PVP–AgNPs is inconsistent. According to Ribeiro et al. [64], El Badawy et al. [58], and Pareek et al. [66], PVP does not give a charge to AgNPs in water at a zeta potential close to zero (−1.4–0.1 mV), while Cornelis et al. [67], Li et al. [50], Wang et al. [68], and Sharonova et al. [69] reported that PVP–AgNPs are negatively charged. According to Cornelis et al. [48], the charge on the surface of PVP–AgNPs is due to the incomplete coverage of the nanoparticles by PVP.

The zeta potential of uncoated AgNPs depends on solution pH and decreases from a positive value at a low pH to a negative value at a high pH, but the surface charge of AgNPs completely covered with PVP chains is not pH-dependent.

The type of stabilization also affects aggregation, and sterically (PVP-) and electrosterically (BEPI-) coated AgNPs are less prone to aggregation than electrostatically (H_2_- and CIT-) coated AgNPs [62].

In addition, the method used to synthesize coated AgNPs has a significant effect on stability and aggregation, and a different CCC was observed for CIT–AgNPs obtained by reducing Ag^+^ to Ag^0^ and then adding CIT solution (CCC ~300 mM NaCl) compared to cleaning an original stock suspension of AgNPs and resuspending it in citrate solution (CCC ~50 mM) [70].

#### 2.2.2. Effect of Solution Properties on AgNPs

The size of AgNPs depends on the pH of the solution and decreases with increasing pH. Therefore, less aggregation and greater particle stability are observed at higher pH [71]. CIT–AgNPs aggregate in an acidic environment (pH 3) [13], but at pH 6–9, they are stable [72,73], while BSA-AgNPs are stable over a wider pH range (2, 3, 7, and 10, except pH 4 and 10) [73]. The effect of pH on the stability of AgNPs may be due to the speciation of citric acid or may be related to the steric effect of BSA. The pH of the solution affects the speciation of citric acid (HOC(COOH)(CH_2_COOH)_2_—H_3_L), and the dominant species at pH < 3 is free acid, but at pH 6 to 9, two species, such as the citrate ion -L^−3^ and the monohydrogen citrate ion -HL^2−^, increase the stability of AgNPs. BSA capping of AgNPs results in steric repulsion and increases colloidal stability over a wide range of concentrations.

Increasing the ionic strength enhances aggregation and adsorption forces. The ionic strength affects the stability of the diffuse layer in the electrical double layer. As the ionic strength increases, compression of the electrostatic double layer occurs, facilitating particle association by reducing particle–particle and particle–surface repulsion [74,75] and promoting agglomeration and increasing retention.

The minimum electrolyte concentration that causes nanoparticles to aggregate has been termed the critical coagulation concentration (CCC). Higher CCC values indicate greater colloidal stability and lower aggregation ability. CCC values are dependent on pH, salt type, cation valency, type of AgNP coating, and acid–base characteristics of the surface.

El Badawy et al. [58] observed the effect of ionic strength on the aggregation of AgNPs (uncoated, CIT-, and NaBH_4_^−^) at a pH above 7, and for PVP–AgNPs, no effect of ionic strength on aggregation occurred due to the type of coating. Sterically PVP-coated AgNPs (CCC = 111.5 mM NaCl) showed higher stability than electrostatically CIT-coated AgNPs (CCC = 47.6 mM NaCl) due to the steric repulsion of the large and non-polymeric groups [54].

AgNPs are generally more likely to aggregate or agglomerate in higher saline solutions [45]. Therefore, they are mobile in an aqueous environment because the typical monovalent salt concentration of fresh water (<10 mM) is much lower than the CCC of AgNPs. AgNPs were more stable in monovalent than in divalent salt, because the CCC for CIT–AgNPs in monovalent electrolytes (NaCl) was observed at 47.6 mM, while in CaCl_2_ and MgCl_2_ electrolytes, it was observed at 2.1 mM and 2.7 mM, respectively [29,76].

The aggregation rate of CIT–AgNPs increased in the presence of dissolved oxygen in solution [77], but the presence of dissolved organic matter inhibited the aggregation of AgNPs [76]. The influence of dissolved organic matter on the aggregation of AgNPs is dependent on structure of the molecules and its molecular weight, including the ratios of non-polar aliphatic, aromatic, and phenolic carbons. Non-polar aliphatic carbons enhance steric repulsion, resulting in the formation of smaller aggregates (0–200 nm), but aromatic and phenolic carbons form aggregates larger than 200 nm [78]. Functional groups (carboxylic and phenolic) play a role in regulating the reduction rate.

#### 2.2.3. Transformation of AgNPs in Aerobic and Anaerobic Systems

One of the most important steps in the transformation of AgNPs, which affects their environmental fate and toxicity, is the release (oxidative dissolution) of Ag^+^ ions in aerobic systems, represented in Equation (1) [79,80,81]:

AgNP^0^ + 1/4O_2_ + H^+^_aq_ = Ag^+^ + 1/2H_2_O. (1)

During dissolution Ag^+^ ions are released from the surface of the particle and migrate through the electrical double layer into the solution [82]. Approximately 10–17% of AgNPs (30–50 nm) oxidize to ions [83]. Oxidized Ag^+^ ions can be adsorbed on surfaces of AgNPs. According to Li et al. [63], the presence of cations in solution favors the oxidation of Ag, as they can displace Ag^+^ ions from the particle surface and thus enhance oxidation.

Particle size, concentration, temperature, dissolved oxygen concentration, presence of ligands, pH, and ionic strength also affect the dissolution of AgNPs [13,84]. The results of thestudy indicated that Ag^+^ ions have a tendency to participate in redox and complexation reactions [85].

Smaller AgNPs (5 nm) tend to dissolve more easily than larger ones (50 nm) [53] and dissolve more quickly in aqueous solution due to a higher surface area, higher density of active sites, and/or due to a lower redox potential, and thus their higher reactivity than large particles [86]. On the other hand, large particles and high concentrations of AgNPs increase aggregation, reduce the surface area and the available reactive sites of AgNPs to oxygen, and inhibit the dissolution rate of AgNPs [80,87]. Dissolution may disappear as the particle size increases [13,85].

The type of AgNP coating and the presence of ligands in solution also affects the dissolution of nanoparticles. CIT–AgNPs had a faster dissolution rate than PVP–AgNPs [88], but in contrast, according to Pareek et al. [66], PVP–AgNPs had greater release of Ag ions than charged coatings. Sigg and Lindauer [89] observed an initial increase in Ag dissolution in the presence of cysteine, while little effect on AgNP dissolution was observed in the presence of fulvic acid (up to 15 mg/L).

The dissolution of AgNPs in the presence of Cl^−^ ions is strongly dependent on the Cl/Ag molar ratio [63,80,90]. At low Cl/Ag ratios, the presence of Cl^−^ ions significantly reduces or inhibits the dissolution of AgNPs by forming AgCl on the surface of the nanoparticles. At high Cl/Ag ratios, increased dissolution of AgNPs due to the formation of the soluble species AgCl_x_^(x−1)−^ was observed [91], and the Ag release rate increased in a short time (<10 h). Ag ions released from AgNPs in the presence of Cl^−^ ions are more favorable in the aerobic system, because in the anaerobic system, sulfidation process of AgNPs occurs by reaction of Ag^+^ with HS- or S^2−^ ions [92].

Depending on the concentration of sulfide, two types of sulfidation of AgNPs can occur: direct and indirect sulfidation. Direct sulfidation occurs in the presence of high concentrations of sulfide ions based on the following reactions:

4AgNP + O_2_ + 2H_2_S → 2Ag_2_SNP + 2H_2_O (2)

or 4AgNP + O_2_ + 2HS^−^ → 2Ag_2_SNP + 2OH^−^ (3)

Indirect sulfidation occurs at low sulfide concentrations, where AgNPs first dissolve Ag and then react with the sulfide ions to form Ag_2_SNPs:

4AgNP + O_2_ + 2H_2_O → 4Ag^+^ + 4OH^−^ (4)

2Ag^+^ + OH^−^ + HS^−^ → 2AgSNP + H_2_O (5)

The sulfidation rate of AgNPs decreases with increasing nanoparticle size [90]. Despite the extremely low solubility of Ag_2_S, traces of dissolved Ag may be released into the aqueous phase.

High concentrations of AgNPs reduce the dissolution of nanoparticles, because at higher concentrations, aggregation occurs more rapidly and inhibits dissolution [13,77].

The dissolution rate of AgNPs also depends on the pH of the solution and increases with decreasing pH [13,85]. In acidic environments, AgNPs dissolve rapidly, indicating that protons played an important role in the process, but in alkaline conditions, the dissolution of AgNPs is slowed [93]. AgNPs also dissolve at higher temperatures [85]. AgNP oxidation depends on both dissolved oxygen and pH, while AgNP dissolution increases with an increase in either. The degree of influence of pH on particle properties is greater than that of dissolved oxygen concentration [85,93].

## 3. Soil Properties Determining Their Adsorption Capacity of Ag

**Figure 2:**
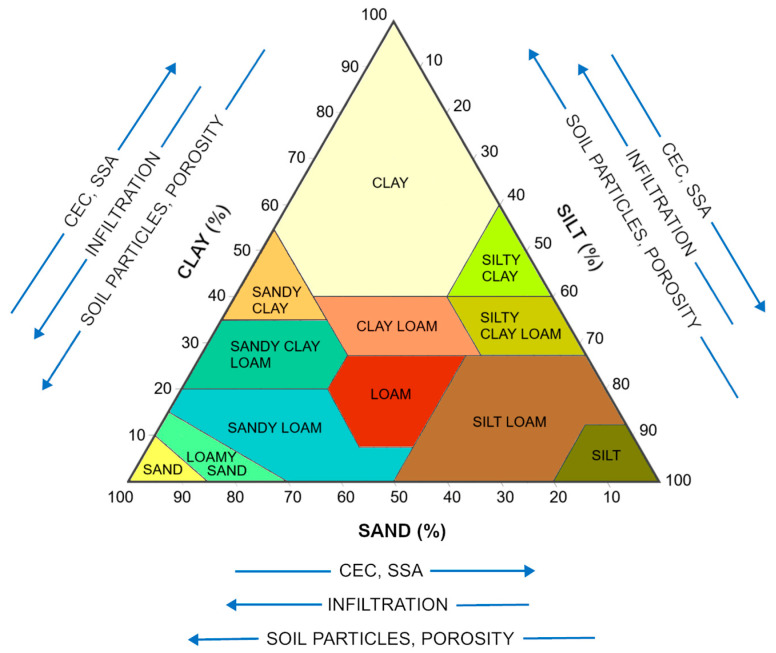
Effect of soil composition on soil properties [94,95].

Soils are sinks/repositories for Ag, which is mainly derived from sewage sludge fertilization. Soils are a highly complex mix of minerals (clay minerals, Fe, Mn oxides, quartz, carbonates) and soil organic matter (SOM) and consist of solid, aqueous, and air phases, with significant amounts of natural colloidal/particulate matter. Air and solution occupy the pore spaces in soils. The composition of the solid phase and the proportions of the components affect the physical properties of the soil, including structure, texture, surface area (SSA), porosity, and pore distribution, but the colloidal fraction affects physicochemical properties such as cation exchange capacity (CEC), anion exchange capacity (AEC), pH and the point of zero charge (pH_PZC_) [94,95] (Figure 2). The physical and physicochemical properties of soils determine the behavior of heavy metals, including their retention and transport.

### 3.1. Main Soil Minerals and Their Properties

Clay minerals and metal oxides are important minerals in the transport and behavior of Ag species in soil. Differences in structure and surface charge result in different properties of the minerals, including SSA, CEC, and pH_PZC_, as shown in Table 1.

#### 3.1.1. Clay Minerals

Clay minerals are present in the clay fraction (<2 μm) of soils and are members of the family of anisotropic phyllosilicates. Their basic structural elements are two-dimensional arrays of silicon–oxygen tetrahedra (T) and aluminum or magnesium–oxygen–hydroxyl octahedrons (O). This group of minerals contains:

- 1:1-type (TO-type) clay minerals, with kaolinite as the main mineral (Al_4_[Si_4_O_10_](OH)_8_)). These sheets are strongly bonded together by shared oxygen ions and form layers. The layers are bonded face to face (silica face–alumina face) by hydrogen bonding between the oxygen of the T sheet and the hydroxyl of the O sheet of the next layer [96]. Kaolinite is characterized by relatively low ion exchange capacity and non-swelling properties due to the lack of net charge in the layers and hydrogen bonding between the layers.
- 2:1-type (TOT-type) clay minerals, with the smectite group with montmorillonite as the main mineral ([(Na,Ca)_0.33_(Al,Mg)_2_(Si_4_O_10_)(OH)_2_·*n*H_2_O]), and illite ([(Al_2_)(Si_4-x_Al_x_)O_10_(OH)_2_·K_1−x_]). Isomorphic substitution in tetrahedral (Al^3+^ instead of Si^4+^) or in octahedral (Mg^2+^ instead of Al^3+^) sheets generates negative charge of the structure, which is compensated by hydrated exchanging cations (Na^+^, K^+^, Ca^2+^). In aqueous solutions, water is intercalated into the interlamellar space of montmorillonite, causing the minerals to expand. Illite is a non-expansive 2:1 phyllosilicate with dehydrated K^+^ ions in the collapsed interlayer.

Clay minerals such as 2:1 have a higher net negative charge and CEC and larger specific surface area (SSA) and swelling effect than other minerals (Table 1).

The main active centers in clay minerals responsible for their adsorption capacity are:

- Exchangeable cations in the interlayer space of smectite;
- Planar sites with permanent charge on the interlayer surfaces (pH-independent) in smectites;
- Edge sites with variable charge on the external surfaces due to proton adsorption/desorption (pH-dependent) in smectites, illite and kaolinite [97].

#### 3.1.2. Metal Oxides

Metal oxides are ubiquitous in soil and have a major influence on soil properties. In soil, iron (Fe) minerals are present as a separate fraction of oxides, (oxyhydr)oxides, and hydroxides (such as hematite α-Fe_2_O_3_, maghemite γ-Fe_2_O_3_, magnetite Fe_3_O_4_, ferrihydrite Fe_1.55_O_1.66_(OH)_1.34_, goethite α-FeO(OH), lepidocrocite γ-FeO(OH)), and/or are associated with a mineral-associated organic matter or clay minerals by adsorption or coprecipitation and form organic–mineral complexes [98,99]. They are reactive in neutral and acid soils. Iron (oxyhydr)oxides are characterized by large surface areas (up to 800 m^2^/g) and high pH_PZC_ values in the 8–9 pH range [100]. However, under natural conditions, their pH_PZC_ may be lower due to the chemisorption of silicate and phosphate anions by Fe oxides [101].

**Table 1:** Physicochemical properties of clay minerals, (oxyhydr)oxides of Fe and Mn, and humic substances [94,95,102].

Soil Components	Physicochemical Properties		
	CEC (cmol_+_/kg)	SSA (m^2^/g)	pH_PZC_
Clay minerals:			
kaolinite	3–15	5–40	2–4
illite	10–40	10–100	3.5–5.0
smectite	80–150	400–800	2.0–4.5
chlorite	10–40	10–55	3.0–4.7
Fe (oxyhydr)oxides	10	800	8.0–9.0
Mn (oxyhydr)oxides	35	15	2
Humic substances	480–1400	800–900	2.5–3.0

During the adsorption process, the surface hydroxyl groups are exchanged by ligands (L) according to the following reactions:

FeOH + L^−^ ↔ FeL + OH^−^, (6)

(FeOH)_2_ +L^2−^ ↔ Fe_2_L + 2OH^−^. (7)

Therefore, organic matter (OC) can be adsorbed onto Fe oxides by the carboxyl and phenolic surface functional groups [102,103], and soil organic carbon content is strongly correlated with Fe oxide content. Kang and Xing [104] found aliphatic fractions of organic carbon adsorbed by Fe hydroxides (such as goethite), but according to Kaiser [105] and Chekli et al. [106], aromatic fractions and organic carboxyl groups have a strong affinity for Fe oxides. The maximum adsorption capacity of Fe oxides for OC was 0.14 g OC/g Fe oxide, and about an order of magnitude higher than that of montmorillonite—0.011 g OC/g Mt [107].

The adsorption of anions on Fe oxides decreases with increasing pH [108], but at higher pH, cations can be adsorbed on Fe oxides according to the following reactions:

FeOH + Me^z+^ ↔ FeOMe^z−1^ + H^+^, (8)

(FeOH)_2_ + Me^z+^ ↔ (FeO)_2_Me^z−1^ + 2H^+^. (9)

In soils, manganese (Mn) occurs as exchangeable ions, oxides, organic forms, and as a component of ferro-magnesian silicate minerals. Mn^2+^ is similar in size to Mg^2+^ and Fe^2+^ ions, and can replace these elements in silicate minerals and Fe oxides. Mn oxides behave differently from Fe oxides in the presence of water. In birnessite (MnO_2_-nH_2_O), the dominant species are MnO_2_^−^ and MnOH^0^, whereas in goethite, these are FeOH_2_^+^ and FeOH^0^. The point of zero net charge for birnessite is pH 2 and for goethite pH 8.4, indicating that at pH 4, they have a net negative or net positive charge, respectively [102].

### 3.2. Soil Organic Matter

The stability and retention of soil organic matter (SOM) depends on the processes of decomposition and mineralization. Over 90% of SOM is associated with colloidal clay minerals, which protect SOM from degradation in the soil [109,110]. Depending on the soil stability, there are:

- A stable fraction, mainly OM associated with mineral phases [110];
- A physically labile fraction associated with free OM aggregates [111].

Dissolved organic matter (DOM), including humic acids (HAs) and fulvic acids (FAs), plays an important role in the behavior of contaminants and their interactions with soil particles. The main differences between FA and HA are molecular weight, surface functional carboxyl and phenolic groups, oxygen content, and acidity [112,113,114], which affect their different adsorption capacities [115]. Sulfur groups on humic matter are also the most important binding sites for Ag^+^ ions in soil systems.

The interactions between Ag^+^/AgNPs and mineral and organic surfaces, including adsorption–desorption to/from the soil particle surface and ion exchange with smectite-exchangeable ions, dissolution–precipitation, transformation, and heteroaggregation control the transport of AgNPs through soils [9,67,116,117,118].

## 4. Behavior of Ag in Soils

### 4.1. Batch and Column Method in Laboratory Study of Ag–Soil Interactions

The geochemistry of AgNPs in soils is complex due to the chemical transformation between AgNPs and Ag^+^ [119]. The interaction of Ag with soil particles depends on a number of geochemical factors, such as clay mineral [119,120] and SOM content, as well as the properties of Ag and the physicochemical properties of the solution [121]. There are a number of variables related to both soil and AgNP properties that need to be considered when studying soil–Ag interactions. Therefore, fundamental studies are carried out under laboratory conditions.

Adsorption parameters and the effect of various factors are mainly studied under static conditions (batch method) or dynamic conditions (continuous flow column method). The nature of the contact between the adsorbate and the adsorbent in a column method is very different from that in batch method. The batch method is often used to estimate of contaminant behavior in saturated soils and aquifer sediments under equilibrium. It provides information on the maximum adsorption capacity of the solid phase and the binding mechanism of the contaminants.

In the dynamic system, the liquid/solid ratio (L/S ratio) is much lower and is determined by the pore solution volume in the minerals under non-equilibrium conditions and simulating saturated or vadose flow conditions. In the presence of macropores whose size exceeds the size of heteroaggregates, rapid infiltration of AgNPs into soil occurs [122,123].

The system demonstrated the role of the colloidal fraction in the transport of metal ions, which is limited by two main mechanisms: (i) straining, where nanoparticles are retained in smaller pore volumes, and (ii) physicochemical processes, where nanoparticles are bound by the solid phase [122]. Contaminant adsorption patterns are presented as cumulative curves showing total adsorbed metal load, retention profile (RP), and breakthrough curve (BTC—C_t_/C_0_ vs. V/V_0_ or time, where C_t_—total Ag concentration, C_0_—input Ag concentration, V—volume of solution, V_0_—retention capacity of soil) [123].

In general, the results of the batch method indicate the retention of Ag by the solid phase, while the dynamic method indicates the potential for transport, including the colloidal fraction, and shows a potential risk of Ag migration to the groundwater [22].

### 4.2. Effect of Soil Composition on Ag^+^ and AgNP Retention

The role of soil composition in the uptake of Ag is investigated by both static and dynamic conditions.

In soil, Ag can react with both soil solution and solid phases. Ag adsorption on natural Australian soils [48], loams with different organic matter content [24], calcareous sandy loams and loams [124], and loamy sands and silty loams [33] showed that it depends on soil composition and its properties.

#### 4.2.1. Binding Ag Forms by the Solid Phase of Soils

Principal component analysis (PCA) between the maximum adsorption (Q_max_) of Ag and soil physicochemical properties [67,68], and multiple regression analyses showed that Ag retention, depending on its form, was significantly positively correlated with Al/Mn or Fe oxides and SOM content, as well as with CEC.

Adsorption capacity of soil for Ag^+^ ions is higher than for AgNPs [30,120], except Fe (oxyhydr)oxide-rich soil. Ag^+^ ions can be bound to pure clay minerals by the following mechanisms [35,125,126,127]:

- Cation exchange in the interlayer space of smectites and at the permanent negative charge of external basal surfaces of smectites forming outer sphere complexes, according to the reaction:

–Surface^2−^–Ca^2+^ + 2Ag^+^ + 4(H_2_O) → Surface^2−^–2Ag(H_2_O)_2_^+^ + Ca^2+^ (10)

- Formation of the Ag hydroxyl surface complex of the cation and silanol (Si–OH) or aluminol (Al_2_–OH) groups at the crystal edges of clay minerals such as montmorillonite, illite, and kaolinite forming inner sphere complexes, according to the reaction:

–Si–O^−^ + Ag^+^ → –Si–OAg or –Al_2_–O^−^ + Ag^+^ → –Al_2_–OAg (11)

**Figure 3:**
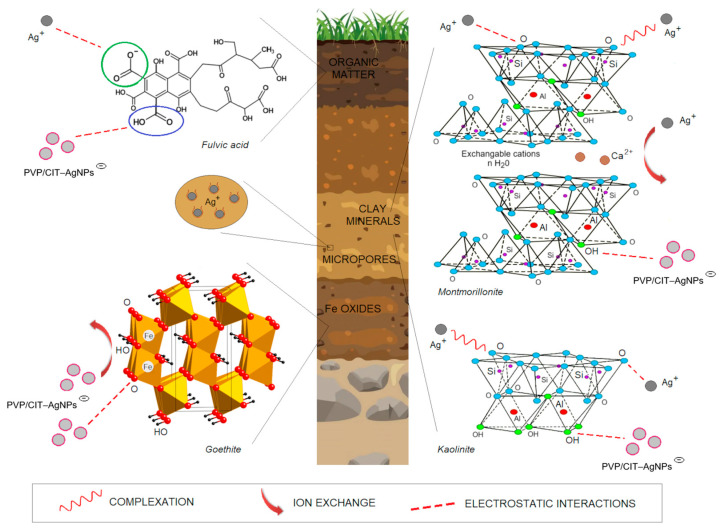
Ag forms binding on the main soil components.

Only silanol groups in clay minerals bound the Ag^+^ ions at pH 4–8, due to the negative charge of the silica tetrahedral face at pH > 4, but the aluminol octahedral face was negatively charged at pH >8 [128]. In contrast, under environmental conditions, the aluminol groups in clay minerals play an important role in the binding of AgNPs [129], but negatively charged silica surfaces cause repulsion of AgNPs [130] (Figure 3).

Torrent et al. [95] confirmed the important role of ion exchange in the binding of Ag^+^ ions in batch tests with five different agricultural soils from the Mediterranean region, in which the clay soil had a much higher adsorption capacity for Ag^+^ ions than the sandy soil. Due to the great number of negatively charged surface sites, ionic Ag^+^ is also well adsorbed in soils with high clay mineral and organic matter (OM) content by electrostatic attraction [131]. Ag^+^ ions (1.26 Å) can also enter many micropores of clay minerals (4.01 nm), whereas AgNPs cannot (>10 nm), due to the much larger size of AgNPs. The retention of Ag in soil pores (pore straining) is a physical process that increases the adsorption of Ag^+^ ions in soils.

Clay minerals and organic matter also play an important role in the binding of AgNPs. Arable soils with higher content of clay minerals and OM (C_org_ 0.1–7.0%, clay content 1–59%) have higher median distribution coefficients for AgNPs than soils with lower content (C_org_ 0.04–2.5%, clay content 0.01–39%): 589 (range 50–2511 L/kg) and 260 L/kg (range 0.6–2391 L/kg), respectively [14].

The retention of negatively charged PVP/CIT–AgNPs differs from Ag^+^ cations due to their charge and interactions with soil particles such that electrostatic attraction or repulsion can occur between Ag forms and the surface of clay minerals, SOM, and Fe oxides, depending on their surface charge.

AgNPs are bound rapidly by electrostatic attraction between negatively charged nanoparticles (CIT-, PVP-) and positively charged edges of clay minerals, so the mineral charge pH dependence controls the retention of AgNPs [48,132,133], whereas according to Hoppe et al. [120], the dominant binding mechanism of AgNPs was heteroaggregation.

The results of adsorption experiments of sterically stabilized AgNM-300k on temperate arable soil samples from northern Germany showed that soils with clay content >202 mg/g had a high uptake of AgNM-300k. The highest risk of long-term release of AgNPs exists when AgNPs are applied to low-clay (<130 mg/g), slightly acidic agricultural soils [120].

Clay soil with smaller particles bound larger amounts of AgNPs compared to sandy loam soil, due to increased physicochemical filtration resulting from the high specific surface area and the presence of adsorption centers on the clay mineral surface [33].

Positively charged Fe oxides provide very good adsorption sites for negatively charged AgNPs (CIT-, PVP-). A study on the adsorption of PVP–AgNPs (zeta potential of −29.3 mV) on soil surfaces (0–20 cm) from five geographical regions of China (eastern, southern, central, northern, and northeastern) [50] showed electrostatic attraction between PVP–AgNPs and Fe oxides, indicating that it is an important process in soils, as shown by Cornelis et al. [132] and Cornelis et al. [67]. Multiple regression analysis of Q_max_ with soil properties showed that total free Fe oxides (Fe_DCB_—dithionite–citrate–bicarbonate extracted) are favorable sites for PVP–AgNP retention [120]. Wang et al. [68] reported that in the case of Ultisol, goethite was main binding site for uptake of PVP–AgNPs. The results of Kyziol-Komosinska et al. [127] on adsorption of bare AgNPs and Ag^+^ ions on ferrihydrite confirmed the electrostatic attraction between AgNPs and Fe (oxyhydr)oxide surfaces and the electrostatic repulsion between Ag^+^ ions and (oxyhydr)oxide surfaces, resulting in higher adsorption of AgNPs than of Ag^+^ ions at higher concentrations (>25 mg/L) (Figure 3).

Despite the large adsorption capacity of Fe oxides, a high degree of Ag_2_SNP dissolution is observed in the pore water of iron-rich acidic soils, ranging from 47.1% to 61.7% [50]. Fe(III) can react rapidly and displace Ag^+^ ions from Ag_2_SNPs due to the much lower K_sp_ of Fe_2_S_3_ (1.4 × 10^−88^) compared to Ag_2_S (K_sp_ = 8 × 10^−51^). This shows that soils with low pH and high Fe content can result in high levels of Ag_dis_ (up to 980 μg/L) and higher bioavailability. In addition, some ligands (e.g., methionine—an α-amino acid containing a carboxyl group and an amino group without a thiol group, thiosulfate, or citrate), as well as elevated Cl^−^ ions in soils can increase Ag_2_SNP dissolution, whereas humic acids and thiol-containing ligands effectively inhibit this process [134,135]. These processes cannot be ignored when studying the behavior of Ag in soils with different characteristics. In contrast, Mn oxides can bind Ag^+^ ions by electrostatic attraction under ambient conditions.

In dynamic conditions, clay minerals and Fe oxides/hydroxides may also be present in the colloidal fraction, as they are relatively mobile and may be involved in the transport of AgNPs in the environment.

The AgNP uptake/mobility in calcareous soils showed that AgNP transport may be affected by surface interactions with the carbonates, and in neutral/alkaline calcareous soils (pH 7.0–8.5), their transport is limited [124]. Calcareous soils contain high concentrations of active Ca^2+^ cations in the pore solution, which increase the ionic strength of the solution. According to Rahmatpour et al. [124], the divalent cation Ca^2+^ may affect the transport of AgNPs by creating nanoscale chemical heterogeneity on the solid surface, which can neutralize or reverse the surface charge at specific locations and/or induce cation bridging between nanoparticles and centers on the soil surface.

The behavior of AgNPs in the soil is also influenced by the particulate SOM. SOM plays an important role in Ag adsorption [136,137] by electrostatic and steric interactions, reduction ofAg^+^ ions [125,138,139,140], inhibition of AgNP oxidation [85], and mobility of AgNPs due to fine texture (silt and clay) and high CEC [24,124]. A study of Ag adsorption on three soils from central New York State with textures ranging from sandy loam (7% clay) to silty loam (21% and 26% clay) and a peaty muck soil rich in organic matter (27% total organic carbon) and two oxides (ferrihydrite and Mn oxide) also showed that OM content could be a dominant factor in Ag adsorption, e.g., strong complexation of Ag by DOM [35]. Organic matter also increases the uptake of AgNPs on sand particles. Ebeling et al. [141] showed a fivefold higher adsorption capacity of wetland soils (sandy loam—sand—71%, silt—23%, clay—6%, C—332.60 g/kg) than silty loam soils (sand—19%, silty—63%, clay—18%, C—19.85 g/kg) (1000 mg/kg and 200 mg/kg, respectively). The results showed that constructed wetlands could be used as sinks for silver nanoparticles, removing them from the wastewater.

Ag^+^ ions are bound by organic matter under alkaline conditions [48,121] via electrostatic and hydration interactions and complexation [30], resulting in lower Ag concentrations in the liquid phase [119] (Figure 3).

#### 4.2.2. Binding of Ag Forms by Dissolved Organic Matter

A decrease in the release of Ag^+^ ions in a soil solution containing DOM (Suwannee River fulvic acid) than in a soil solution without DOM due to complexation with Ag^+^ ions by metal-binding functional groups was observed by Cornelis et al. [67] and also described by Philippe et al. [115]. One of the retention mechanisms of Ag^+^ ions is the precipitation of metallic Ag by reduction by humic substances and lignite [125,126].

Due to the negative charge of humic substances at environmental pH, adsorption will make the entire AgNP–humic substance heteroaggregate negatively charged [142]. DOM adsorption on AgNP surface increases the electrostatic repulsion forces between particles and/or increases the surface hydrophobicity and can stabilize AgNPs against aggregation, thus increasing the transport and mobility of AgNPs [48,143]. The presence of humic acids in both soil and soil solution also increases the mobility of AgNPs due to their ability to stabilize AgNPs in suspension. The humic acid layer formed around AgNPs can modify the chemical interaction between soil and AgNPs, but does not have a significant effect on mechanical deformation, because AgNPs form a stable suspension even in the absence of humic acid [144]. Humic acid and fulvic acid (from the Suwannee River) could be adsorbed on the AgNP surface and block the reaction sites with oxygen, decreasing the dissolution of CIT–AgNPs in acetate buffer at pH 5.6 [85].

The Ag adsorption process can affect particle properties in a number of ways. Interactions between Ag^+^ ions and/or AgNPs and dissolved humic substances can lead to the formation of: (i) relatively stable AgNPs colloids/agglomerates [34], (ii) spherical HA–Ag complexes [145], and (iii) thin hydrophilic layers on nanoparticles by fulvic acid (with a high charge density), thus reducing steric hindrance [146]. AgNPs and Ag^+^ ions in soil undergo different transformations, as evidenced by different secondary forms, including AgCl, metallic AgNPs, Ag_3_PO_4_, and Ag_2_O.

Nanoparticle properties (e.g., size, coating, and surface charge) affect the behavior of AgNPs [30,120]. Bare AgNPs better adsorbed low-molecular-weight, short-chain non-aromatic DOM than long-chain aromatic DOM [1]. PVP and CIT capping agents increased the mobility of AgNPs by blocking the binding of AgNPs to soil [62,120,130]. The stable forms of AgNPs formed by Ag^+^ reduction in the presence of fulvic acids of DOM can be transported over considerable distances and can also affect the overall bioavailability of AgNPs [138]. The adsorption of OM on AgNPs is non-linear and is well described by the Langmuir adsorption isotherm equation [147,148].

The composition of the DOM also affected the size of the aggregates. AgNPs formed larger aggregates in the presence of hydrophilic DOM than in the presence of hydrophobic DOM due to the higher charge density of hydrophilic DOM. The stabilizing effect of DOM also depends on the initial AgNP concentration and plays a significant role only at higher initial AgNP concentrations [1]. Larger aggregates tend to be retained in the upper layers of the soil, potentially reducing pore size and/or clogging the soil pores [75].

Among the different functional groups in SOM, the sulfur groups play an important role in the binding of Ag by complexation [120]. The sulfide groups have the potential to bind Ag, and according to Li et al. [50], the retained Ag follows Ag_2_SNPs > Ag^+^ > AgNPs. The higher adsorption of Ag_2_SNPs may be due to their larger size, as shown by transmission electron microscopy (TEM) analysis (104.6 ± 18.8 vs. 29.4 ± 4.6 nm).

In addition, contact time is an important parameter in assessing the effect of AgNP charge and the presence of coating groups, and has been shown to delay and retard the extent of particle agglomeration, dissolution, and sedimentation in the short term, whereas this effect becomes less important after longer periods (>6 months) [121,149].

The results show that adsorption of Ag^+^ ions and AgNPs on soils is well described by Langmuir, Freundlich and linear isotherms [30,50,120].

The Langmuir model describes the adsorption of AgNPs and Ag^+^ ions on ten soils from China well [30]. On the other hand, the results of Li et al. [50] showed that only the Freundlich isotherm describes the adsorption of Ag forms on soils well. The K_F_ (an approximate indicator of adsorption capacity) value for AgNP adsorption ranged from 21 to 1408 (mg/kg)·(L/mg)^1/n^, with the highest value for yellow soil, but the K_F_ for Ag^+^ ions adsorption ranged from 13 to 1116 (mg/kg)·(L/mg)^1/n^, with the highest value for paddy soil. Hoppe et al. [120] indicated that the retention of Ag^+^ in soils was also best described by the Freundlich isotherm equation. The K_F_ values were high for most of the soil horizons studied (mean K_F_ = 3144 (mg/kg)·(L/mg)^1/n^). Only acidic sandy subsoils had lower K_F_ values, suggesting that the mobility of Ag^+^ ions in these subsoils was higher than in the organic rich topsoils. The adsorption of AgNM-300k was described by the linear isotherm [120]. The results of the adsorption of bare AgNPs and CIT–AgNPs showed that despite the differences in zeta potential (−36 mV and −59 mV, respectively), the adsorption isotherms were very similar, indicating that zeta potential does not strongly affect the adsorption of AgNPs [1].

### 4.3. Effect of pH–Eh and Ionic Strength of Solution

The behavior of AgNPs in soils is also influenced by the solution properties, such as the pH, the redox potential, the ionic strength and the type of cation, and the concentration of ligands and oxygen [117].

The pH value is one of the most critical factors in Ag speciation and oxidative dissolution of AgNPs and the surface charge of colloidal particles of clay minerals, Fe (oxyhydr)oxides, and humic substances by dissociation of amphoteric functional groups, and coagulation rate [71,150]. On the other hand, the surface charge of soil particles is a parameter that influences the heteroaggregation of AgNPs with soil particles [48]. The retention of PVP–AgNPs was negatively related to pH and EC in the PCAs performed by Cornelis et al. [67] and Wang et al. [68]. At the high pH in AgNPs partially covered by PVP and CIT, AgNPs bind only on surface of Fe oxides by electrostatic interactions [48]. At low pH (<5.1), coated AgNPs tend to destabilize with oxidation of the nanoparticles [120,131], but steric repulsion reduces aggregation of AgNM-300k at moderate pH values. Alkaline soils have a higher potential to bind nanoparticles than acidic soils because the pH of these soils is closer to the pH at which the AgNP surfaces become zero-charged, facilitating nanoparticle aggregation [31].

Adsorption of Ag^+^ ions is also controlled by pH, and at high pH, soils have higher adsorption capacity for Ag^+^ ions, for example, black and red soils in China [151], but under acidic conditions, Ag^+^ has a lower affinity for soil surfaces and therefore are more bioavailable. Furthermore, under alkaline conditions, Ag^+^ ions can be reduced to metallic Ag [152] and bind to clay minerals.

Besides pH, Eh also controls the dissolution of AgNPs in soils [131,153]. The results of Li et al. [119] showed that with increasing Eh, Ag^+^ ion concentrations increased by 1.1–41.0-fold. The distribution of Ag in the soil depends on the depth in the soil profile and indicates that in the presence of oxygen and in the absence of sulfides, it is bound by clay minerals and organic matter, but in deeper soil, in the absence of oxygen, Ag forms sulfide complexes [46,154]. The conversion of AgNPs to Ag_2_S is always incomplete, and the sulfidation process does not prevent the uptake andtoxicity of Ag by organisms, and Ag can accumulate in terrestrial plants and organisms.

In oxic soils, Ag is more weakly bound and the mobility of the Ag^+^ ions is controlled by retention processes. In acid soils, AgCl was also formed, but the oxide (Ag_2_O) and carbonate (Ag_2_CO_3_) forms were rarely found despite Eh soils [155].

The behavior of Ag forms in soil also depends on the ionic strength and valence of ions of the aqueous phase. According to Bae et al. [156], aggregation of AgNPs in soil increased with increasing ionic strength. The increase in AgNP adsorption at high ionic strength is due to the physicochemical properties of the soil surface, such as the electrostatic double layer, but without homoaggregation processes [33].

The type of salt (KNO_3_, NaNO_3_, Mg(NO_3_)_2_ and Ca(NO_3_)_2_) and its concentration in solution play a major role in the behavior of AgNPs in soil [29]. The uptake of PVP–AgNPs by clay soils increased with increasing KNO_3_ and Ca(NO_3_)_2_ concentrations due to increased aggregation of PVP–AgNPs and reduced repulsive interaction between PVP–AgNPs and soil particles [33]. Divalent cations (Ca^2+^ and Mg^2+^) have been shown to be more effective in binding AgNPs than monovalent cations (Na^+^) at the same ionic strength [31], and can increase aggregation and adsorption [157]. In particular, reducing the ionic strength of the solution and/or replacing divalent cations with monovalent cations can cause colloid/nanoparticle release by reducing the depth of the secondary energy minimum [31].

The presence of Ca^2+^ ions in the soil solution decreases the stability of AgNPs despite the presence of DOM, which increases the stability of AgNPs by electrostatic forces and steric effects [1]. In contrast, Ag is immobilized in the presence of Ca ions at high pH values. In the presence of Ca^2+^ ions, bridging occurs between DOM molecules and AgNPs, which can increase AgNP aggregation [158,159]. Ca^2+^ can also cause aggregation between SOM and the organic AgNP coating. In addition, the presence of soil colloids can modify the effect of salt on the behavior of AgNPs. In the co-presence of Ca^2+^ and HA, the aggregation of PVP– and CIT–AgNPs in both sandy loam and clay soils increased compared to the presence of Ca^2+^ alone [144].

The presence of chloride also plays an important role in the retention of AgNPs in the soil, as chloride ions can reduce transport in the soil profile [144]. AgNPs can be coated with a layer of AgCl, or AgCl can precipitate during the release of Ag^+^. In addition, the type of AgNP coating affects the rate of the Ag–Cl reaction. The presence of a citrate coating on the AgNP surface, which acts as a barrier between the chloride and the Ag core of the AgNPs, can slow down the normally very rapid reaction between Cl^−^ ions and AgNPs.

### 4.4. Transport and Mobility of AgNPs in Soil

The transport of AgNPs in different soil types through simulated saturation, near-saturation, and vadose zone condition has been studied under dynamic conditions.

Rahmatpour et al. [124], Sagee et al. [144], He et al. [22], and Braun et al. [33] found that the uptake and mobility of AgNPs in soil also depended on air content in the soil pores, flow rate, and AgNP shape, size and concentration. According to Rahmatpour et al. [124], the degree of saturation and the presence of air under unsaturated conditions had little effect on the transport of AgNPs by calcareous soils, probably due to the high retention of AgNPs in the soils.

AgNP retention increases with decreasing flow rate [144]. Increased retention at lower solution flow rates is due to increased contact time between the solution and soil particles and increased diffusion, leading to trapping of nanoparticles in the pores of soil aggregates. These results are consistent with filtration theory, which predicts increasing retention with decreasing flow rate [160]. At low flow rates, the role of diffusion is much more important than advection.

The results of AgNP transport through loamy sand and silty loam columns [33] under different flow rates were strongly dependent on the mineral composition of the soil. A study of the transport of AgNPs in loamy sand (illite, montmorillonite, kaolinite) showed that retention increases with decreasing flow rate and that AgNPs are mobile in the soil at high flow rates. A 60-fold reduction in flow rate (from 0.20 cm/min to 0.0033 cm/min) resulted in strong retention of AgNPs in the soil, with only 1.79% of the AgNPs passing through the column, due to the increasing effect of diffusion and a negligible effect of advection [33]. In contrast, AgNP transport was completely inhibited in silty clay (illite, chlorite/vermiculite, kaolinite, and swelling illite–smectite minerals), which may be due to increased filtration due to low flow rates and high fine soil content and increased adsorption sites related to the mineral composition of the soil.

AgNP transport depends on the chemical composition of the soil solution. At a flow rate of 0.20 cm/min, the presence of the monovalent cation Na^+^ in the solution reduces AgNP transport only at higher concentrations, whereas the presence of the divalent cation Ca^2+^ affects AgNP transport at the low concentration of 1 mM. Transport of AgNPs through the soil resulted in a slight decrease in nanoparticle size at higher flow rates and a slight increase at lower flow rates [33].

The effect of Ag properties such as particle size (15 and 27.4 nm), surface coating (PVP and CIT) and input nanoparticle concentration (2.5, 5, and 10 mg/L) on AgNP transport and mobility was investigated by He et al. [22] in clay loam: red soil from Yujiang County, Jiangxi, China, in the saturated zone. The maximum adsorption capacity of the soil studied was 199.5 mg/kg for large PVP–AgNPs. The mobility and transport of AgNPs increased with increasing input nanoparticle concentration and nanoparticle size, because smaller particles are more easily retained in the pores, but larger particles are more mobile [117]. In addition, capping agents (PVP and CIT) strongly influence the surface chemistry as well as their mobility and reactivity [65], increasing the mobility and thus the potential risks of AgNP migration to groundwater [22].

The transport and mobility of AgNPs can be analyzed using a numerical model that takes into account the time-dependent and depth-dependent retention. The shape of the breakthrough curves (BTCs) and retention profiles (RPs) depend on the physicochemical properties of the soil and the experimental conditions. According to He et al. [22], the normalized effluent concentration of AgNPs (C/C_0_) increased with increasing input concentration (C_0_), and at higher C_0_, the slope of the BTC was steeper. The concentration dependence of AgNP transport is due to their blocking effect [161], as active sites are rapidly occupied at higher C_0_. The input concentrations of AgNPs also had a significant effect on the shape of the RPs, and non-monotonic distributions of retained AgNPs were observed. The non-monotonic RP is due to time-dependent retention and down-gradient migration of AgNPs, depending on the input concentration. Sagee et al. [144] and Cornelis et al. [48] found that the early breakthrough behavior of AgNPs in soil depends on the soil pore size. This is related to the nature of AgNP transport in natural soils. Large-diameter nanoparticles cannot enter small pores due to their size or repulsive forces. As a result, they are transported through larger pores. Their rate is higher than that of the solvent, which can pass through smaller pores. According to Sagee et al. [144], the BTCs depend on the flow rate. Braun et al. [33] found that BTCs for different flow rates are asymmetric with a late breakthrough, indicating time-dependent blocking behavior. RPs at a high flow rate show a hyperexponential shape, while RPs at a low flow rate were exponential. The exponential distribution of deposited particles with transport distance is compatible with filtration theory.

Rahmatpour et al. [124] observed hyperexponential RPs for calcareous sandy loam and clay soils under saturated and unsaturated conditions and in the presence of Ca^2+^ and K^+^ ions. He et al. [22] found that the coating type of AgNPs affected the retention profiles. The RPs shape was non-monotonic for PVP–AgNP samples, but it was hyperexponential for CIT–AgNPs. The different RP shapes can also be explained by the time-dependent retention of AgNPs. The shape of RPs depends on nanoparticle size. For large AgNPs (27.4 nm), a hyperexponential shape was observed with 81.8% of AgNPs retained near the column inlet (1–3 cm). In contrast, a non-monotonic deposition of small AgNPs (15 nm) was observed with a maximum depth of 8 cm. Larger AgNPs have a higher mass transfer rate from the aqueous phase to the solid phase because they tend to sediment rapidly due to strong gravitational forces [160]. At high flow rates, a hyperexponential RP formed in the columns because of a higher mass transfer rate of AgNP to the collector surface due to the hydrodynamic conditions at the column inlet. In addition, a more hyperexponential RP was observed with higher ionic strength and lower AgNP input concentration.

Modeling analysis of AgNP transport in partially saturated soil suggests that the transport of AgNPs is very well described by a two-site kinetic model with a time-dependent retention function [162]. The study successfully described the experimental BTCs and RPs of AgNPs by using one-species and two-species models.

## 5. Susceptibility to Release of Adsorbed Ag in Soil

In addition to retention, information on the binding strength of the adsorbed metal is required to assess the susceptibility to release, mobility, and bioavailability of the adsorbed Ag forms. Leaching tests (e.g., DIN 38414-S4) and the DTPA method (method with diethylenetriaminepentaacetic acid) [163] and/or sequential chemical extraction are used. The DIN 38414-S4 leaching test [164] provides information on the potential for metal release, but the DTPA leaching method provides information on the bioavailability of metals to plants [165,166]. Sequential chemical extraction procedures (first formulated by Tessier et al. [167] for sediments) have been used to assess the partitioning of metals in environmental samples using a series of chemical extractants to displace and dissolve the soil fraction with metals. For Ag, the sequential extraction procedure developed by Coutris et al. [116] is based on six fractions: E1—exchangeable, E2—weak acid solution, e.g., carbonate bond, E3—easily reducible, Fe/Mn oxides, E4—oxidizable, organic matter, E5—acid digestible with strong mineral acid, E6—residual. The procedure is often preceded by a leaching test—E0 (Table 2). Stages E0–E2 (using inert electrolytes) involve displacement processes that are reversible without breaking chemical bonds, but stages E3–E6 involve irreversible dissolution processes of soil constituents and associated Ag release. The sequential extraction method does not determine the form of Ag in the soil fraction, only the total Ag content.

The mobility of adsorbed Ag depends on the uptake mechanism, and Ag species adsorbed by cation exchange are more susceptible to release than those immobilized by precipitation. According to Benoit et al. [131], high pH can reduce Ag mobility. Moreover, different soil properties such as high clay content strongly influence the mobility of Ag [116,155]. In addition, studies of the effects of soil properties (mineral and organic) and Ag forms (CIT–AgNPs vs. Ag^+^ ions) on Ag mobility indicate that soil properties affect Ag speciation more than the original form of Ag. These results showed that Ag^+^ ions can behave similarly to AgNPs in soil, as these ions are rapidly reduced in contact with soil organic matter, transforming them into nanoparticles and colloids. In contrast, CIT–AgNP complexes reacted more slowly than Ag^+^ ions, and in the initial days after the experiment, the concentration of CIT–AgNPs in the water-leachable fraction was higher than that of Ag^+^ ions, but after 35 days, the amount of Ag leached by water was similar for both forms in the soils.

The results of Kyziol-Komosinska et al. [127] showed that Ag^+^ ions were bound to clay minerals by ion exchange, mainly in the mobile E1–E2 fractions and for montmorillonite E1 ≥ E2, but for kaolinite E2 ≥ E1, uncoated AgNPs were bound in the immobile E4 and E5 fractions. According to Sposito [168], hydrolytic species are preferentially adsorbed on mineral surfaces compared to free cations because they are easier to desolvate than free metal ions.

Ag^+^ ions adsorbed on ferrihydrite were mainly bound in E3 [127], indicating that the presence of ferrihydrite can reduce the mobility of Ag^+^ ions from the soil phase [169].

According to Torrent et al. [95] and Kyziol-Komosinska et al. [127], Ag^+^ ions and AgNPs were bound in the pores of the minerals at low levels of <5% and <0.5%, respectively. In contrast, according to Coutris et al. [116], 21–37% of CIT–AgNPs adsorbed by inorganic soils were bound in a water-extractable form, indicating the mobility of the nanoparticles. These values were much higher than those found by Kyziol-Komosinska et al. [127], and could be due to the clay mineral content, the properties of the AgNPs studied (CIT–AgNPs and bare AgNP forms), and the contact time.

The presence of Ag in the E5 fraction in mineral soils with very low organic matter content is probably due to the dissolution of nanoparticles and not to the oxidation of organic matter. The fourfold higher Ag content in the E5 fraction of Ag^+^-loaded mineral soil may indicate that Ag colloids formed from AgNO_3_ are more readily oxidized than CIT–AgNPs [116].

The E3 fraction was higher in mineral soils than in organic soils because Ag in organic soils is more strongly bound to organic matter than to Mn/Fe oxides [116]. The authors also noted a different distribution of bare AgNPs in soil compared to Ag^+^ ions and CIT–AgNPs. After 70 days of contact, the mobility of Ag from uncoated AgNPs bound to mineral soil was two- and fivefold higher than that of Ag^+^ ions and CIT–AgNPs, respectively. In organic soils, these differences were greater, with the mobility of Ag from uncoated AgNPs being nine- and eightfold higher that of Ag^+^ and CIT–AgNPs, respectively. The authors explained these differences by a slower dissolution rate of uncoated AgNPs compared to AgNO_3_ and CIT–AgNPs, the tendency of AgNPs to aggregate, and the dissolution rate. All forms of silver studied were more mobile in the mineral soil than in the organic rich soil.

Hashimoto et al. [155] suggested that the redox potential of the soil has a significant effect on the behavior and determination of AgNPs and Ag^+^ ion transformation. They showed that under aerobic conditions, the content of AgNPs in the E1 and E2 fractions was lower than Ag^+^ ions, and 88% of the AgNPs added to the soil remained stable after 30 days, while Ag^+^ ions were bound to humic substances and clay minerals. Under anaerobic conditions, 83% of AgNPs added to the soil were converted to Ag_2_S, with a decrease in the E0–E2 fractions, and about 50% of the Ag^+^ ions were converted to metallic Ag and bound to clay minerals. The dissolution of Ag from AgNPs was reduced, due to the formation of Ag_2_S. The dominant reaction of AgNPs under anoxic conditions is sulfidation.

## 6. Conclusions

1. One of the most popular and widely produced nanoparticles is silver nanoparticles (AgNPs), and 20% of nanoproducts on the market contain Ag. With the increasing production and use of AgNPs, they can be released into the water and soil environment during the life cycle of the products through atmospheric deposition, fungicide application, irrigation with wastewater, and application of sewage sludge as fertilizer.
2. In soils, AgNPs can undergo many transformations, including oxidative dissolution, and therefore the behavior of AgNPs and ionic Ag^+^ should be considered in parallel.
3. The behavior of Ag in the soil depends on the properties of the nanoparticles (e.g., nanoparticle size, coating type and surface charge, and the initial amount of Ag).
4. The main binding mechanisms of AgNPs are:
  - Electrostatic attraction between negatively charged AgNPs (CIT–, PVP–) and positively charged Fe oxides;
  - Electrostatic attraction between AgNPs and positively charged aluminol (Al_2_OH) groups on the edges of clay minerals and formation of AgNP complexes with aluminol (Al_2_OH) groups on the edges of clay minerals;
  - Adsorption of AgNPs on OM by electrostatic and steric interactions and reduction in aggregation and inhibition of oxidation of AgNPs in the presence of OM.
5. The main binding mechanisms of Ag^+^ ions are:
  - Cation exchange between Ag^+^ ions and exchangeable cations in the interlayer space of smectite and adsorption onto the permanent negative charge of the outer basal surfaces of smectite and kaolinite;
  - Formation of Ag^+^ hydroxyl surface complexes with silanol (SiOH) groups on the edges of clay minerals;
  - Adsorption of Ag^+^ on OM under alkaline conditions by electrostatic interaction, hydration interaction, and complexation and/or precipitation of metallic Ag;
  - Retention of Ag^+^ in soil pores (pore straining).
6. The highest risk of long-term mobilization of AgNPs exists in soils with low clay content (<130 mg/g) and in slightly acidic conditions.
7. The values of pH and Eh affected the speciation of Ag and charge of mineral surfaces and the adsorption of Ag:
  - At low pH, the nanoparticle coatings tend to destabilize, and oxidation and dissolution of AgNPs and high bioavailability occur;
  - The high pH reduces Ag^+^ mobility;
  - In aerobic systems and in the presence of Cl^−^ ions, formation of AgCl occurs at low Cl/Ag ratios or dissolution of AgNPs occurs at high Cl/Ag. In anaerobic systems, sulfidation occurs.
8. The transport and mobility of AgNPs depend on:
  - The input AgNP concentration and nanoparticle size and the charge of the capping agent.
  - The flow rate of the solution. The retention of AgNPs in the soil increases at lower flow rates due to increased contact time between the solution and solid phase and increased diffusion.
9. Sequential chemical extraction showed that Ag^+^ ions were bound in mobile fractions, whereas AgNPs were bound in immobile fractions. The presence of ferrihydrite can reduce the mobility of Ag^+^ ions from the soil phase.
10. Understanding the transport and behavior of Ag in soil is essential for environmental risk assessment and management of wastes containing Ag.

## Figures and Tables

**Table 2:** Sequential extraction procedure [126].

Stage of Extraction	Fraction	Extractant	Condition		
E0	Water-soluble	Deionized H_2_O	S:L = 1:20, 1 h at 20 °C	→ Mobility	
E1	Exchangeable	1M CH_3_COONH_4_ (pH 7)	S:L = 1:20, 2 h at 20 °C		
E2	Weak acid dissolution (e.g., carbonate bound)	1M CH_3_COONH_4_ + CH_3_COOH (pH 5)	S:L = 1:20, 2 h at 20 °C		
E3	Easily reducible (Fe/Mn oxides)	0.04 M NH_2_OH·HCl + 25% CH_3_COOH (pH 3)	S:L = 1:20, 6 h at 80 °C		
E4	Oxidizable (organic matter)	30% H_2_O_2_ + HNO_3_ (pH 2) then 3.2 M CH_3_COOH at 20% HNO_3_	S:L = 1:20, 5.5 h at 80 °C S:L = 1:5, 0.5 h at 20 °C		
E5	Acid-digestible	7 M HNO_3_	S:L = 1:20, 6 h at 80 °C		
E6	Residual	Total sorption—F0 + F1 + F2 + F3 + F4 + F5			

## Data Availability

No new data were created or analyzed in this study.
